# Influence of aging on deterioration of patients with COVID-19

**DOI:** 10.18632/aging.202136

**Published:** 2020-11-24

**Authors:** Limin Pang, Yi Liu, Maoze Shen, Jujian Ye, Ruirong Chen, Zhien Lan, Zhijian Wu, Yang Guo, Peidong Zhang

**Affiliations:** 1 Department of Cardiology, Heart Center, Zhujiang Hospital, Southern Medical University, Guangzhou, Guangdong, People’s Republic of China; 2 Department of Neurology, Zhujiang Hospital, Southern Medical University, Guangzhou, Guangdong, People’s Republic of China; 3 Department of Cardiology, Boai Hospital of Zhongshan, Southern Medical University, Zhongshan, People’s Republic of China; 4 Department of Internal Medicine, Raoping County People's Hospital, Chaozhou, Guangdong, People’s Republic of China

**Keywords:** aging, COVID-19, GRP78, ACE2, CD147

## Abstract

Aging is an important factor affecting the deterioration of patients with coronavirus disease 2019 (COVID-19). The aging and degeneration of various tissues and organs in the elderly lead to impaired organ function. Underlying conditions such as chronic lung disease, cardiovascular disease, and diabetes in aged patients are associated with higher mortality. Severe acute respiratory syndrome coronavirus 2 (SARS-CoV-2) primarily interacts with the cell surface receptor angiotensin-converting enzyme (ACE) 2 and other accessory proteins such as 78 kDa glucose-regulated protein 78 (GRP78) and CD147. Thus, altered receptor signals in aging and chronic disease play a role in SARS-CoV-2 infection, and are associated with a higher risk of deterioration in different organs. In this review, after a brief introduction to the link between aging and receptors for SARS-CoV-2, we focus on the risk of deterioration in different organs of COVID-19 patients considering aging as the main factor. We further discuss the structural and/or physiological changes in the immune system and organs (lung, heart, kidney, vessels, nerve system), as well as those associated with diabetes, in aging patients, and speculate on the most likely mechanisms underlying the deterioration of COVID-19 patients.

## INTRODUCTION

**Figure 1:**
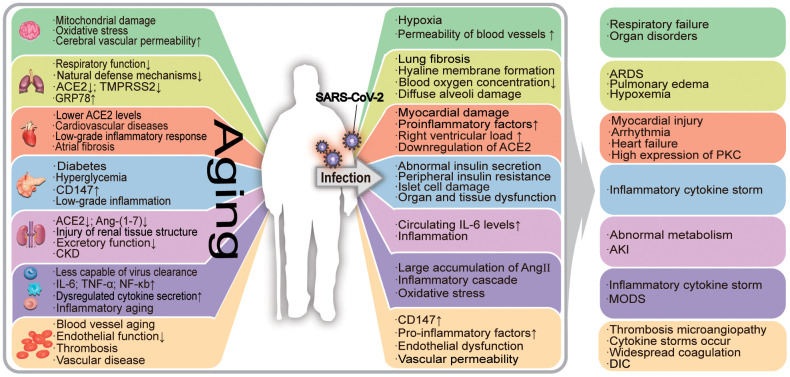
**The effects of aging on the organism and its relationship with COVID-19-associated deterioration.** Aging patients are more susceptible to SARS-CoV-2, which leads to the exacerbation of COVID-19. Aging has a negative impact on the human organ system, causing its functional decline. The aging process is accompanied by a state of low-grade inflammation, and the elderly are more susceptible to SARS-CoV-2 infection. Under the influence of the virus, the damage to infected aging patients is further worsened, resulting in serious secondary diseases. In the figure, "↑" means the number increases, the function or effect is enhanced; "↓" means the number decreases, the function or effect is weakened. ERS: endoplasmic reticulum stress; ACE2: angiotensin-converting enzyme 2; TMPRSS2: transmembrane protease serine 2; ADAM17: a disintegrin and metalloprotease 17; ARDS: acute respiratory distress syndrome; AKI: acute kidney injury; PKC: protein kinase C; ROS: reactive oxygen species; CKD: chronic kidney disease; DIC: disseminated intravascular coagulation.

Biological aging in humans is characterized by genomic instability, telomere attrition, epigenetic alterations, loss of proteostasis, dysregulated nutrient sensing, mitochondrial dysfunction, cellular senescence, stem cell exhaustion, and altered intercellular communication. These abnormal changes in the body lead to age-related damage [1, 2]. The accumulation of aging-related damage leads to increased morbidity and mortality among the elderly [3]. Aging lowers the human body’s resistance to pathogens and impairs the subsequent immune responses. In the case of severe acute respiratory syndrome coronavirus 2 (SARS-CoV-2) pneumonia, the damages caused by human aging are closely related to the deterioration of the condition of the patients. Aging adults are more likely to have underlying comorbidities and are therefore at greater risk of deterioration of coronavirus disease-2019 (COVID-19) [4]. In Figure 1, we have summarized the association between COVID-19 and aging in various organs.

### The ongoing age-related COVID-19 pandemic

**Figure 2:**
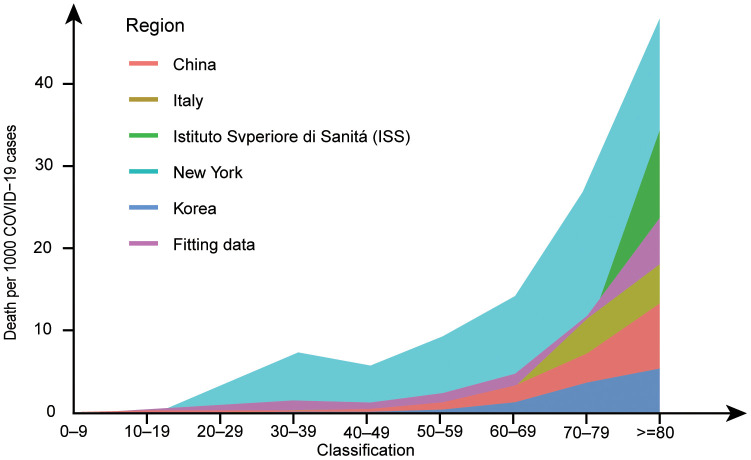
**Deaths per 1,000 COVID−19 cases by age group in major epidemic areas.** Data from references [6, 101–104] have been amalgamated with the above data to fit the new data. After standardizing the data for each group, the probability of death at each age was fitted and multiplied by 1,000. The ggplot2 package in R version 4.0.0 was used to draw a line graph. Marine green represents New York, red represents China, blue represents South Korea, yellow represents Italy, grass green represents ISS, and purple represents the fitting data. In the line graph, the number of deaths per 1,000 COVID-19 patients increases with age.

Following the initial reports of COVID-19 in December 2019, this disease rapidly became a pandemic and a major global public health concern. COVID-19 is an infectious disease caused by SARS-CoV-2. From a global perspective, the situation remains bleak as this pandemic has concentrated medical attention on treating people with COVID-19. There is a widespread lack of immunity to this emerging pathogen, and the world’s population is largely susceptible to this virus. Epidemiological studies have shown that COVID-19 is less common in children than in older adults, especially those with underlying comorbidities. Moreover, middle-aged and elderly people are the most affected by this disease, while elderly patients and those with underlying diseases have more acute and shorter courses of the disease [5–13]. We integrated the data from several reports and found that the ratio of deaths to infections was higher in the elderly than in the other age groups (Figure 2). Comorbidities, including chronic lung disease, cardiovascular disease, chronic kidney disease (CKD), and diabetes, are also associated with higher mortality in older patients [9, 14, 15].

### The link between virus receptors and aging

SARS-CoV-2 belongs to the same beta coronavirus subgroup that also comprises severe acute respiratory syndrome coronavirus (SARS-CoV) and Middle East respiratory syndrome coronavirus (MARS-CoV). Viruses in this group have an affinity for angiotensin-converting enzyme 2 (ACE2) and the auxiliary molecule, transmembrane serine protease (TMPRSS2) [16–18]. Studies have shown that SARS-CoV-2 invades the human body via the ACE2 receptor. Moreover, the affinity of SARS-CoV-2 for ACE2 is ten-fold higher than that of SARS-CoV. The receptor-binding domain (RBD) of the S1 subunit of the viral Spike protein binds to ACE2 to invade target cells [19–21]. In coronavirus infection, ACE2 bound by the coronavirus will be cleaved by a disintegrin and metalloprotease 17 (ADAM17, also called TNF-α converting enzyme, TACE), thereby reducing the concentration of ACE2 at the cell surface [22]. Studies have shown that the overall expression of ACE2 in the human body is age- and tissue-dependent. The concentration of ACE2, as well as that of other receptors associated with SARS-CoV-2 infection, is higher in adults than in children [23], which may result in a higher rate of infection. However, ACE2 expression in cells of pulmonary, renal, and cardiovascular tissue has been demonstrated to decline with age [24–26]. The function of ACE2 is to convert angiotensin (Ang) II into Ang-(1–7) and increase the level of superoxide dismutase (SOD), which can reduce cell peroxidation-related damage [27, 28]. Ang-(1–7) can reduce Ang II concentrations and antagonize its activity. Meanwhile, plasma ACE2 activity reportedly increases with age [29], which can increase production of Ang-(1–7). ACE2 shedding through ADAM17-mediated cleavage may be associated with increasing Ang II levels, as well as the modulation of the renin-angiotensin system (RAS) in cardiovascular and chronic diseases [30–32].

Recent evidence has suggested that SARS-CoV-2 can also bind to CD147 and 78-kDa glucose-regulated protein (GRP78) expressed on the cell surface [33, 34]; the levels and functions of both proteins may also be related to aging. Blocking CD147 has an inhibitory effect on SARS-CoV-2, suggesting that CD147 may promote SARS-CoV-2 invasion [35, 36]. The levels of CD147, known as a key target for malaria treatment, can increase with age, thereby also increasing the number of targets for invasion by the virus [37, 38]. Cell surface-localized GRP78 mediates endoplasmic reticulum stress (ERS) and can help to activate transcription factors and maintain cell homeostasis. Because viral infection and nutrition deprivation can stimulate the ERS-associated unfolded protein response (UPR) [39], SARS-CoV-2 infection will likely lead to increased GRP78 concentrations, as recently reported [40]. However, GRP78 expression can decrease with aging, thereby lowering the ability of the endoplasmic reticulum to clear misfolded proteins, which is associated with poor prognosis in older COVID-19 patients [41].

### The aging immune system and low-grade inflammation

In the elderly, COVID-19 is more likely to lead to complications, including the outbreak of a “cytokine storm”, acute respiratory distress syndrome (ARDS), multiple organ dysfunction syndrome (MODS), and eventually even death. Deceased patients typically display a sustained increase in interleukin (IL)-6, D-dimer, lactate dehydrogenase, and serum ferritin levels [14, 42], suggesting that patients suffer severe inflammation and pathological damage before the end of life.

An aging immune system is less capable of virus clearance. Thymus glands, the spleen, and lymph nodes undergo degeneration in aging, which interferes with the normal differentiation of T cells, phagocyte viability, and the secretion of natural killer (NK) cells [43, 44]. In the elderly, an aging immune system leads to poor recognition and elimination of viruses and cancer cells and an overall decline in the immune response of normal cells [45].

Cytokine storms are more likely to occur in the elderly. According to the theory of immunologic dissonance, the imbalance of pro-inflammatory and anti-inflammatory forces in the elderly leads to destructive immune dysregulation [46, 47]. Reduced ACE2 expression during aging increases ovalbumin-induced eosinophil, lymphocyte, and neutrophil recruitment [48] and leads to dysregulated cytokine secretion, which ultimately results in damage to various organs. On the one hand, owing to the immaturity of the proliferative pool, the absolute number of neutrophils is significantly lower in newborns than in adults. This is manifested by the high expression of adhesion molecules (e.g., P-selectin) and the increased chemokine production by resident inflammatory cells [49], decreased reactivity, and increased extravasation. On the other hand, the absolute number of neutrophils is thought to be related to aging. Aging leads to increased expression of IL-6 and TNF-α in the body, which aggravates the inflammatory response [50], and can also lead to the upregulation of nuclear factor-kappa B (NF-κB), the main regulator of IL-6 and IL-1β, thereby altering the levels of these cytokines. Viral invasion aggravates inflammation, and inflammatory factors continue to accumulate while the body's immune system fails to clear these inflammatory factors in a timely manner [51]. The aging process is accompanied by a state of low-grade inflammation. The serum level of inflammatory mediators is significantly higher in the elderly than in the young. Low-grade inflammation can induce changes in several signaling pathways, and, importantly, can also lead to the deterioration of COVID-19 and age-related diseases [52, 53]. One feature of chronic inflammation due to aging is a change in the Ang II signaling pathway. Factors downstream of Ang II, such as IL-6 and TNF-α, promote the occurrence and development of inflammation. After SARS-CoV-2 invades the human body, the downregulation of ACE2, when combined with aging, will cause a large accumulation of Ang II. Simultaneously, Ang II can integrate mechanisms such as reactive oxygen species (ROS) formation, leading to a vicious cycle of cellular aging [27].

An increase in the levels of Ang II disrupts the balance between ROS production and removal, which further intensifies oxidative stress [27]. ROS production through NOX2, a member of the NADPH oxidase (NOX) family of enzymes, has been demonstrated to play a key role in lung injury [54]. The ROS and hydrogen peroxide produced during oxidative stress can lead to tissue injury and irreversible damage to macromolecular proteins. ROS is indirectly responsible for the body's proinflammatory response and the accumulation of proinflammatory factors, which leads to a worsening of the overall condition and ultimately triggers cytokine storms and MODS.

### Lung aging and COVID-19

One of the most obvious characteristics of COVID-19 infection is viral pneumonia. Out of a group of 1,591 patients, 88% needed respiratory support [10], similar to the results reported for another group of patients [55]. In another cohort comprising 1,099 cases, 3.4% had ARDS and >59% had abnormal pulmonary computed tomography (CT) findings [9]. The clinical features of ARDS, also known as acute respiratory failure (ARF), comprise altered respiratory system mechanics and hypoxemia. Middle-aged and elderly people are more likely to develop ARF and show a higher rate of intensive care unit (ICU) admittance than younger patients.

The effect of SARS-CoV-2 on ACE2 can easily reduce lung function in the elderly. Reduced ACE2 expression due to SARS-CoV-2 infection further reduces ACE2 in the lungs of the elderly, but without affecting ACE content [22, 56]. Without ACE2, the conversion of Ang II into Ang-(1–7) is reduced, thereby allowing Ang-(1–7) to exert a protective effect against pulmonary edema, reduce pulmonary vascular resistance, resist tissue damage, and reduce myeloperoxidase content in the lungs [48]. High levels of Ang II hyperactivate the Ang II type 1 receptor (AT1R) in the lungs, contributing to the contraction of bronchial smooth muscle cells and increased pulmonary capillary permeability, resulting in a dry cough, pulmonary edema, and difficulty breathing [28]. The expression of GRP78 protein decreases with age, which affects lung tissue repair and aggravates viral infection-associated damage. In the aged lung, the GRP78 level decreases while ERS increases, which causes lung fibrosis [57]. Apoptosis occurs more readily in older patients than in younger ones under high levels of ERS induced by virus infection [58]. In SARS-CoV-2 infection, abnormal apoptosis can disrupt the repair and remodeling of lung tissue and disturb endoplasmic reticulum homeostasis, which aggravates the irreversible damage to the lungs and leads to unfavorable prognosis.

The respiratory function and natural defense mechanisms are lowered in the aging lung, which disrupts its ability to clear the virus and regulate hypoxic adaptation. The influence of advanced age on many aspects of lung immunity, including a series of structural and physiological changes to the lungs, is reflected in cell dysfunction and changes in innate immune system signals [59]. Studies have shown that many patients with severe COVID-19 present with basic pulmonary diseases. In the elderly, the lung is likely to develop an emphysema-like phenotype, which is also observed in chronic obstructive pulmonary disease (COPD) [60]. The effects of oxidative stress and mitochondrial DNA damage are obvious in the aging lung tissue, and lead to high levels of apoptosis in lung cells in COPD patients [61]. The severity of COVID-19 is markedly increased in COPD patients [9] and may suggest that an emphysema-like phenotype and other age-associated structural changes can exacerbate COVID-19. In patients aged >60 years, there is an evident decline in the functions of the respiratory immune barrier, especially alveolar phagocytosis, ventilation, tracheal epithelial ciliary movement, and cough reflex, resulting in poor virus clearance [59]. In terms of respiratory function, the contraction and relaxation of the diaphragm muscles, which are closely related to ventilatory activities, can help to relieve the symptoms of hypoxia that are associated with COVID-19. However, the function of the diaphragm muscle is weakened with aging, resulting in abnormalities in excitation–contraction coupling, organizational structure, and metabolism [62]. Aged lungs have a reduced capacity to alleviate hypoxia. Respiration will be affected if the nerve center is infected with the virus, as will diaphragm function [63]. Osteoporosis in the elderly also influences the shape of the chest, limiting lung contraction [60].

A heightened inflammatory response in aging will exacerbate COVID-19. Ground-glass opacity (GGO) and bilateral lung plaque shadow are commonly seen in chest CT scans of COVID-19 patients. The scope of lesions expands with disease aggravation. The severe period can quickly progress to a wide range of diffuse pulmonary change, and pleural effusion, so the lungs show large white areas in CT images [64]. In autopsies of COVID-19 patients, the lungs showed diffuse alveoli damage and hyaline membrane formation, and a large number of viscous secretions overflowing from the alveolar cells, displaying the pathological characteristics of ARDS [34]. A severe inflammatory reaction in the lung is indicated by neutrophil accumulation, causing deconstruction and edema of the celiac capillaries, which then damage the gas-blood barrier [65]. During aging, lungs tend to have low-grade inflammation, such as an increased neutrophil response [49, 65]. Inflammatory changes associated with aging can lead to increased lung infection rates in older patients [59]. In contrast, neutrophils flow into the lungs and the associated inflammatory mediators are less reactive in newborn and juvenile subjects. The functions of lung dendritic cells, NK cells, macrophages, and neutrophils decline with age and reduce the body’s defenses against external pathogenic microorganisms [59].

In summary, neutrophils accumulate in the lungs of elderly patients and cannot be easily discharged, which not only damages the innate immune response of the alveoli but also promotes inflammation. The number of alveoli cells declines in this condition, causing hyaline membrane formation, pulmonary edema, and other lung injuries. The respiratory disorders caused by COVID-19 lead to a sharp reduction in blood oxygen concentration and aggravate organ damage.

### Heart aging and COVID-19

Worldwide, a large proportion of COVID-19 patients have cardiovascular disease. In China, one study reported that 15% and 2.5% of patients with COVID-19 presented with hypertension and cardiovascular disease, respectively [9]; in New York, the same complications accounted for 57% and 11% of patients, respectively [6]. In a region of Italy, 49% of patients had hypertension and 21% had coronary heart disease, and the elderly with hypertension had a higher mortality rate with respect to ICU deaths [10]. Elderly patients have a more acute presentation. Some COVID-19 patients have acute heart injury [8] or show inflammation-related cell infiltration in the myocardium [66]. Deceased patients with myocardial injury show elevated levels of troponin [67].

The impact of SARS-CoV-2 on ACE2 interferes with the regulation of the RAS in heart aging. As previously mentioned, SARS-CoV-2 infection can result in the downregulation of ACE2. In aging, high levels of ACE2 can effectively protect against Ang II-induced cardiac fibrosis and hypertrophy [68]. However, the hearts of older subjects have lower ACE2 levels and fibroblast activation and transition to a myofibroblast phenotype [69]. Some COVID-19 patients typically show increased IL-6 and C-reactive protein levels before death [14, 55], indicative of the progress of inflammation in the COVID-19 heart. The release of proinflammatory factors can cause heart damage [32], exacerbating myocardial cell apoptosis through various signaling pathways [69]. Cumulative heart injury due to hypertension, valvular heart disease, and heart failure in the elderly can lead to a low-grade inflammatory response. Low-grade inflammation maintains RAS activation and oxidative stress. As the anti-peroxidation effect of ACE2 can protect the heart blood vessels [70], the lack of ACE2 in the myocardium due to aging enhances oxidative damage and promotes inflammatory responses in the injured area. An imbalance between ACE and ACE2 exacerbates cellular damage among elderly patients when the myocardium undergoes an inflammatory reaction in COVID-19.

Atrial fibrosis occurs as a consequence of cardiovascular diseases during aging and can cause atrial overload and stretching [69]. The downregulation of ACE2 is associated with the occurrence of cardiac fibrosis. Fibrosis-induced changes to the heart structure and the associated increase in the number of fibroblasts can lead to more frequent arrhythmias and their progression to persistent or permanent arrhythmias. Several studies have mentioned the gradually increasing risk of arrhythmia in aging [71, 72]. In COVID-19 patients, the hyaline membrane can lead to a sharp drop in blood oxygen content [55] and puts extra strain on the heart. Cardiomyocyte energy metabolism can be damaged by the decrease in blood oxygen content. Right ventricular load is further increased because of edema and hyaline membrane formation in the aged lung, which increases pulmonary vascular resistance. If COVID-19 patients have an underlying heart condition and hypertension that have already damaged the cardiomyocytes, heart failure or arrhythmia can easily and rapidly occur in an already damaged lung.

Aging can decrease the tolerance to COVID-19 treatment in an aged heart. The use of chloroquine or hydroxychloroquine leads to an extension of the Q-T interval [73]. This finding indicates that the use of these drugs will also increase the risk of arrhythmia. Considering the decline that occurs in the pump function of the heart in the elderly, special attention should be paid to changes in the electrocardiography reads of these patients when selecting COVID-19 drug treatment. In patients with pneumonia, right heart failure and elevated levels of NT-ProBNP (a marker of heart failure) [74] are suggestive of a worsening of the condition and are associated with a significant increase in the fatality rate [75], which should also be considered when administering COVID-19 treatment.

### Kidney aging and COVID-19

Patients with severe COVID-19 are more likely to have kidney injury. One study showed that approximately 15% of COVID-19 patients had acute kidney injury (AKI) [14]. Aging, comorbidities, and medical intervention predispose elderly patients to AKI [76]. In SARS-CoV infections, viral particles were found in the kidney, suggesting that the virus can infect this organ [77]. Given that SARS-CoV-2 has a higher affinity for humans than SARS-CoV [17], the kidneys are more likely to be a target of infection for SARS-CoV-2, resulting in AKI in elderly patients.

Elderly patients are considerably more likely to have an AKI in COVID-19 because the renal tubular function can be damaged by free radicals produced in the aging body [78]. The kidney tissue structure displays a reduced number of healthy nephrons, changes to the tubulointerstitium, thickening of the glomerular basement membrane, and increased glomerulosclerosis. Increased Ang II activity in the elderly leads to the accumulation of proinflammatory cells, thereby exacerbating the injury to renal tubular endothelial cells. In aging, the kidney lacks ACE2 and Ang-(1–7), resulting in glomerular sclerosis, which limits the role of proximal tubular cells and podocytes in resisting oxidative stress and cell proliferation [32, 78]. A cytokine storm caused by COVID-19 increases circulating IL-6 levels in the body; hence, the proinflammatory effects, combined with a lack of renal ACE2 protection, leads to a severe functional injury to the kidneys.

CKD (affected 5% of the 5,700 COVID-19 patients in the New York study [6]), hypertension, and heart failure are all comorbidities that increase the risk of AKI and are commonly coexisting illnesses in COVID-19 patients [9]. Patients with CKD usually have increased levels of AT1R in peripheral leukocytes [79], indicating that continuous Ang II activation can exacerbate kidney injury [80]. Uremic conditions due to CKD promote the excessive activation of monocyte ACE levels and inhibition of ACE2, thereby promoting endothelial adhesion and migration and possibly also atherosclerosis development [79]. The changes in blood flow in renal atherosclerosis reduce kidney elasticity and thicken the tunica intima, leading to prerenal AKI [76]. Renin secreted by glomerular cells is a key hormone for the production of Ang II, and glomerular cells are regulated by the RAS. As previously mentioned, coronavirus infection can reduce the concentrations of ACE2 on the cell surface, including in renal cells, which can lead to abnormal metabolism in the kidneys, and even in the whole body.

Because the excretory function of the kidney is lowered in aging, its capacity to excrete drugs and harmful metabolites is reduced. Electrolyte disturbances, such as hypokalemia, often occur as a result of fever, gastrointestinal symptoms, and reduced ACE2 levels in COVID-19 [81]. The aging kidney can undergo morphological and anatomical changes and display reduced filtration capacity [78], leading to increased drug-associated toxicity in the body. The disease process promotes an unstable internal environment and is more likely to result in a serious acid–base imbalance in the elderly, and even result in severe conditions. In terms of treatment, clinicians should be cautious when administering drugs that are excreted through the kidneys, especially in patients with a preexisting kidney injury.

### Aging and vascular injury in COVID-19

Patients with COVID-19 have a high incidence of vasculitis and thrombosis in the lungs [82]. Some can also have Kawasaki disease [83], petechiae, tiny bruises, and/or transient livedoid eruptions in the skin [84]. Biochemical indicators such as prothrombin time, activated partial thromboplastin time, fibrinogen, and D-dimer are significantly increased or decreased [55], indicating that SARS-CoV-2 also affects the blood system and induces disseminated intravascular coagulation (DIC).

Vascular disease, a significant problem in the elderly, can easily cause serious endothelial damage during COVID-19 evolution. Aging arteries are characterized by changes in microRNA expression patterns, autophagy, smooth muscle cell migration and proliferation, and dynamic calcification [68], while vasculopathy is associated with increased ROS production, oxidative stress and deficiency of peroxidase [85]. Increased blood pressure, elevated blood sugar, obesity, low-density lipoprotein cholesterol, and sodium intake in the elderly can all influence endothelial function through oxidative stress and inflammatory disorders. The arterial walls develop atherosclerotic plaques and have increased rigidity and stiffness, leading to a reduction in arterial compliance [68]. Pathological changes during blood vessel aging play a role in the initial stages of DIC. Endothelial cells function to promote vasodilation, fibrinolysis, and suppress aggregation, and can inhibit the formation of thrombosis [28]. Endothelial cell dysfunction can facilitate the occurrence of microthrombosis [68, 86, 87]. Because ACE2 can increase the activity of endothelial cells, in the absence of ACE2 in COVID-19 and aging, Ang II activation causes smooth muscle contraction, induce high expression of proinflammatory factors, promote vascular contraction and endothelial dysfunction, all of which contribute to increased vascular permeability and eventually become predisposing factors for DIC [28]. A proinflammatory status in aging leads to enhanced plasma concentrations of inflammatory proteins [68]. When suffering from a COVID-19-associated cytokine storm, the anti-inflammatory properties of aging arteries decrease; this, when coupled with the presence of SARS-CoV-2 virus within the endothelial cells [82], is more likely to cause widespread coagulation throughout the body.

CD147, is expressed on the surface of blood cells [36]. Increased CD147 expression can occur during platelet activation or some inflammation-related responses [37], suggesting that the expression of CD147 may increase after SARS-CoV-2 infection. The combination of these factors can render the elderly more prone to systemic edema and congestion when suffering from COVID-19-related pneumonia, and is more likely to cause functional changes in organs with rich blood flow such as the kidneys, lungs, heart, and brain.

### Aging of the nervous system and COVID-19

Many patients with COVID-19 have clear neurological symptoms such as cerebrovascular disease, unconsciousness, and skeletal muscle tremors [88], and at least one study reported the presence of SARS-CoV-2 mRNA in the cerebrospinal fluid of patients [89].

Elderly patients are more likely to be infected by SARS-CoV-2 in the central nervous system. Two other coronaviruses—SARS-CoV and MARS-CoV—display neuronal tropism [90, 91], which suggests that SARS-CoV-2 may have the same feature. Increased Ang II levels resulting from inflammatory responses such as the cytokine storm will lead to an increase in the permeability of blood vessels in the brain, increasing the possibility that the virus may reach the brain. Oxidative stress resulting from aging and inflammation also upregulates Ang II expression in the brain [92]. Thus, increased Ang II levels during aging can enlarge the site and scope of the infection among the elderly. When SARS-CoV-2 invades the brain stem, respiratory failure and organ disorders can become much more common symptoms of COVID-19.

Nerve aging and chronic disease are associated with the production of the proinflammatory factors IL-6 and IL-1β by microglia. The most common manifestations of deteriorating COVID-19 pneumonia in elderly patients are severe ARDS and MODS, both of which display increased circulating IL-6 levels. The dependence of the brain on a high oxygen concentration, unsaturated fatty acids, and strong mitochondrial metabolism lead to obvious high oxidative stress- and mitochondrial-related damage as a result of aging and microglial priming [93]. Brain damage in hypoxia due to COVID-19 leads to excessive activation of microglia and release of proinflammatory factors [93], which further aggravates the brain injury. SARS-CoV invasion of the brain was reported to increase the density of microglia [91]. This suggests that SARS-CoV-2 infection can also promote the overactivation of microglia, leading to a more severe inflammatory response, and even an “intracerebral storm” of inflammatory factors. SARS-CoV-2 infection may further reduce the availability of ERS-associated proteins, as well as that of GRP78 and other chaperone proteins, in the elderly brain. This will cause apoptosis [58] and exacerbate brain damage.

Patients with COVID-19 often have increased bronchial secretions [42], and aspiration pneumonia may also be a possible cause for disease aggravation. Blockages caused by the secretions can lead to severe hypoxemia and lung tissue injury. In COVID-19 patients, hypoxia and the impact of SARS-CoV-2 on neurons reduce nerve responses, whereby the bronchial secreta cannot be effectively discharged through coughing and swallowing reflexes. Several neurodegenerative diseases can lead to slower reflexes in elderly patients when compared with young patients, which means that the nerve responses in the elderly can be more easily reduced. A recent study [94] reported that the use of an ACE inhibitor can alleviate pneumonia in stroke patients by reducing the effects of Ang II on the brain as well as substance P and bradykinin metabolism. These effects can enhance the cough reflex, prevent aspiration, and ultimately reduce the risk of pneumonia in the elderly; however, further evidence is required with respect to the risks and benefits of ACE inhibitor application.

### Diabetes in the elderly and susceptibility to COVID-19

In the elderly, aging is associated with diabetes, which can lead to chronic diseases such as hypertension, atherosclerosis, and kidney injury [95]. Zhou et al. reported a 19% incidence rate of diabetes among COVID-19 patients [14], similar to that reported in other studies [6, 9, 10]. This suggests that diabetes is an underlying risk of developing severe COVID-19.

Diabetes is one of the most commonly diagnosed age-related comorbidities, and promotes inflammation and SARS-CoV-2 infection in the aging body. Hyperglycemia decreases proximal tubule ACE2 by activating the process of ACE2 shedding through ADAM17-mediated cleavage described in the previous [30]. The virus can attach to the shedding ACE2 and spread throughout the body. Hyperglycemia-induced ACE2 glycosylation can also lead to a decline in ACE2 function. Meanwhile, in type 2 diabetes, insulin can activate vascular ACE activity in vascular smooth muscle cells, and inhibit angiotensinogen and renin expression. In aging, the diabetes can enhance the sensitivity of local cells to inflammatory mediators and activate toll-like receptors to cause insulin resistance [95]. Hyperglycemia can enhance Ang II concentrations in cardiomyocytes and fibroblasts, which promotes the release of inflammatory mediators. Hyperglycemia also induces increased expression of CD147 [36], leading to an increased probability of SARS-CoV-2 infection. Because of the close link between matrix metalloproteinases (MMPs) and CD147 [37], hyperglycemia can influence the migration of monocytes and the function of fibroblasts, as well as the release of TNF-α, vascular endothelial growth factor (VEGF), and IL-1β. Elevated expression of MMPs can also promote pulmonary inflammation, causing a cytokine storm, which is difficult to suppress, and result in systemic injury.

Concomitantly, diabetes and islet aging can affect the whole body in patients with deteriorating COVID-19. Strong ACE2 protein immunostaining was observed in the islet cells of patients with SARS-CoV infection: the higher the level of immunostaining, the greater the damage to the organs [96]. This suggests that the pancreas can also be attacked and damaged by SARS-CoV-2. This damage to pancreatic islet cells leads to abnormal insulin secretion, exacerbates the endocrine disorder in patients, and aggravates the damage to other organs [97]. COVID-19 may further lead to organ and tissue dysfunction because of the increased risk of cardiovascular disease and stroke resulting from diabetic nephropathy and diabetes [98]. High levels of insulin exert growth stimulatory effects on vascular cells and increase the formation of atherosclerotic plaques [99]. In a review of the relationship between GRP78 and aging [58], insulin secretion and insulin-like growth factor (IGF-1)-mediated regulation of GRP78 appeared to enhance the adaptive capacity of the UPR under ERS conditions. Aging can cause impaired autophagy in islet cells, which can lead to impaired β-cell function. The accumulation of misfolded and aggregated proteins can activate the UPR [100]. GRP78 also plays a role in glucose homeostasis and has antiobesity properties. If the activity of GRP78 changes under the influence of COVID-19, the steady-state of insulin and ERS will change, resulting in an ERS-induced decline in immune resistance.

### Summary

The SARS-CoV-2 virus outbreak is ongoing since December 2019. Numerous studies have attempted to clarify the relevant characteristics of the virus. However, the exact mechanisms underlying the high mortality rate of patients with chronic diseases and older patients requires further in-depth analysis. To date, the vaccine for the SARS-Cov-2 is still in development, and there is no specific drugs targeting the virus. Therefore, in clinical practice, it is necessary to pay close attention to changes in the condition of elderly patients, make accurate judgments, and prevent cytokine storms and multiple organ failure in as timely a manner as possible. Timely prevention and correct treatment at the time of disease onset can prevent disease progression and the deterioration of patients, thereby reducing mortality.
